# The Detection of Water Flow in Rectangular Microchannels by Terahertz Time Domain Spectroscopy

**DOI:** 10.3390/s17102330

**Published:** 2017-10-13

**Authors:** Yan Song, Kun Zhao, Jian Zuo, Cuicui Wang, Yizhang Li, Xinyang Miao, Xiaojing Zhao

**Affiliations:** 1 State Key Laboratory of Petroleum Resources and Prospecting, China University of Petroleum, Beijing 102249, China; 2016316011@student.cup.edu.cn (Y.S.); 2015316009@student.cup.edu.cn (Y.L.); 2016316010@student.cup.edu.cn (X.M.); 2 Beijing Key Laboratory of Optical Detection Technology for Oil and Gas, China University of Petroleum, Beijing 102249, China; 3 Department of Physics, Key Laboratory of Terahertz Optoelectronics, Ministry of Education, Capital Normal University, Beijing 100048, China; zuoj@cnu.edu.cn (J.Z.); wangcuicui@cnu.edu.cn (C.W.); 2150602045@cnu.edu.cn (X.Z.)

**Keywords:** water flow, THz-TDS, rectangular microchannel, flow-rate, pressure drop, flow resistance

## Abstract

Flow characteristics of water were tested in a rectangular microchannel for Reynolds number (*Re*) between 0 and 446 by terahertz time domain spectroscopy (THz-TDS). Output THz peak trough intensities and the calculated absorbances of the flow were analyzed theoretically. The results show a rapid change for *Re* < 250 and a slow change as *Re* increases, which is caused by the early transition from laminar to transition flow beginning nearly at *Re* = 250. Then this finding is confirmed in the plot of the flow resistant. Our results demonstrate that the THz-TDS could be a valuable tool to monitor and character the flow performance in microscale structures.

## 1. Introduction

The emergence of micro-electro-mechanical systems has attracted significant interest in the field of microscale devices. During the last few years, because of the immense potential of micro systems such as higher accuracy, lower power and lower cost, micromachined fluidic systems have had an important impact on a wide variety of areas such as medicine, chemical analysis, bioengineering, molecular separation and other industries. However, due to the higher surface to volume ratio caused by the smaller typical length, the surface force as well as microchannel wall wettability has great influence on the fluid movement; thus, there will be significant departure of flow characteristics from the conventional flow as fluid is induced to flow through the microchannel [1,2]. As such, in order to predict the flow performance in such micro devices accurately, it is necessary to understand the fluid flow characteristics on the microscale.

In fact, the design and control of microfluidic devices require the fundamental understanding of flow characteristics such as flow pattern transition and pressure loss. As reported by early investigators, the flow characteristics in microchannels are different from that in the normal situation described by the Navier-Stokes equations [3]. It has been found that the transition of the flow pattern from laminar to turbulent flow might take place much earlier than that for flow going through conventional larger-sized channels [4,5]. Other investigations have supported the earlier findings and have served to illustrate that the flow characteristics are strongly affected by the surface roughness [6,7].

Vicente et al. employed water and ethylene glycol as working fluids in dimpled tubes to measure the laminar and transitional flow characteristics. They reported a relatively low critical Reynolds number (*Re*) down to 1400 owing to the surface roughness and then proposed the correlation for the prediction of critical *Re* in dimpled tubes. They declared that dimple height of dimpled tubes was the main factor affecting the hydraulic behavior of the flow [8]. Similarly, Kandlikar et al., investigated the flow of distilled water through small circular tubes with various hydraulic diameters and surface roughness. They stated that the surface roughness had a dramatic effect on the pressure drop for the smaller diameter tube and the critical *Re* for smaller rough tube was much lower than 2300 [9]. Li et al., applied microscopic particle image velocimetry (microPIV) to measure instantaneous velocity fields in a polydimethylsiloxane (PDMS) microchannel at various *Re*. By analyzing the great variation caused by velocity fluctuations in the individual velocity fields, the calculated velocity fluctuations could be used to predict the critical *Re*. They found laminar flow ceased at *Re* of 1535, and fully turbulent flow was achieved at 2630 < *Re* < 2853, both of which were lower than classical results [10].

Optical measurements of the microfluidic flow have been proposed recently. Lauri et al., measured the flow velocity profile in a capillary with two Doppler optical coherence tomography (DOCT) systems, and depth scanning was also achieved by moving the whole measurement system with the reference mirror fixed [11]. Lucchetta et al., utilized an optofluidic device consisting of a Bragg grating written on a soft wall to measure flow rate in a microfluidic channel. They used diffraction of a white-light probe beam as detecting wave and established a simple theoretical model for the response time of the diffracted signal to determine the flow rates [12]. However, there is still little research on understanding the characteristics and affecting factors of the flow in microchannels by terahertz time-domain spectroscopy (THz-TDS). Preliminary studies on the flow pattern and the slip phenomenon for oil-water two phase flow in macroscale pipes based on THz-TDS have been made previously [13,14]. Owing to the sensitivity of THz waves to the fluctuations of water dipole moments occurring on the picosecond (ps) timescale, THz technology can detect the subtle changes of water [15,16], so attempts have been made to predict the flow pattern transition of single phase flow in a rectangular microchannel using a method based on THz-TDS in this work. The flow-rates, the pressure drops and the THz signals of water in microchannel were measured and analyzed and the interaction between THz wave and flow pattern was analyzed. We then calculated the flow resistances of the flow, which verified the experimental analysis further. The results of this study demonstrate the potential of THz-TDS for microfluidic studies.

## 2. Materials and Methods

**Figure 1:**
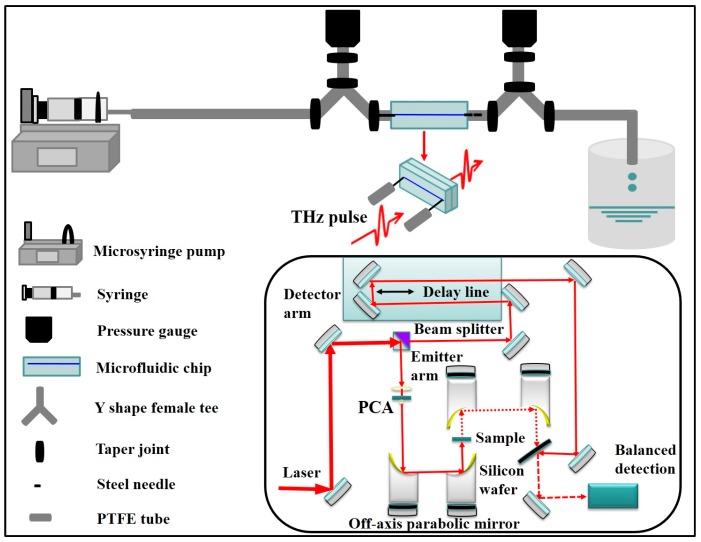
Sketch of the experiment arrangement for detection of deionized water flowing in microchannel with transmission THz-TDS and setup of transmission THz-TDS systems (inset).

A schematic diagram of the experimental apparatus is shown in Figure 1. The experiments were carried out with a single channel plastic microfluidic chip which was fabricated on a 29 mm × 11 mm × 5.6 mm PDMS plate. The microdevice was fabricated by the molding method which includes two steps, namely the production of a SU-8 glue mold and casting molding of the PDMS chip. There are four sub-steps to get the SU-8 glue mold. The first step is to design the microchip shape with CAD and transfer it to the chrome plate. The second step is to cast a thin layer of SU-8 photoresist on silicon wafer and then carry out high temperature treatment. The next step is exposure and cure, in which the chrome plate is used as the mask and the cured SU-8 negative photoresist is exposed with the lithography machine. The last step is to get the SU-8 glue mold by dipping the silicon wafer in the developer. The casting molding of PDMS chip is described in brief. First, PDMS prepolymer should be prepared. The ratio of PDMS and curing agent is 10:1, and the prepolymer needs to be degassed for 10 min. Then the degassed PDMS prepolymer is poured onto the SU-8 mold, and is heated in a 75 °C drying chamber for about 1 h. After that, the cured PDMS is removed from the SU-8 glue mold. When the two PDMS plates are put in the plasma cleaning apparatus, it is worthwhile to note that the two sides being bonded should remain upwards, and the plates should be taken out 2 min after the appearance of violet light and then the two plates should be fitted within 30 s. The fourth step is to place the fitted chip in the oven at 80 °C for 1 h. The obtained microdevice, as shown in Figure 1. It consisted of a capillary and two stainless steel needles whose dimensions are 0.5 mm × 0.8 mm × 15 mm. The cross-section of the microchannel is rectangular, with dimensions of 200 μm × 50 μm × 20 mm (*W* × *H* × *L*), resulting in the hydraulic diameter *D_H_* = 80 μm:

(1) $$D_{H}=4S/(2H+2W)$$

where *S* is the cross-sectional area and the draw ratio *L*/*D_H_* is equal to 250, thus the entrance effects can be ignored [17]. The digital pressure gauges were connected to the microdevice by tubes in the experiments. All the connection tubes are 1.6 mm outer diameter and 0.6 mm inner diameter PTFE (Polytetrafluoroethylene) tubes. The working fluid was deionized water and it was introduced to the horizontal rectangular microchannel by digital injection pump. The flow velocity of the water was increased gradually at the range of 0–5.58 m/s. When setting a new flow-rate, the system ran for at least 5 min until the flow remained stable with very few fluctuations in the pressure drop readings. The density and viscosity of the water are 998 kg/m^3^ and 1.01 mPa·s respectively.

The behavior of deionized water in the microchannel was followed by a conventional transmission THz-TDS whose bandwidth is beyond 2.5 THz. The detailed transmission principal of THz-TDS system is depicted in Figure 1. The femtosecond pulses are provided by a mode-locked Ti: sapphire laser (Mai Tai) whose center wavelength and average power are 790 nm and 200 mW respectively. The amplitude and phase information of the sample are obtained by THz-TDS in a coherent way. The femtosecond laser pulse is divided into two beams, namely pump beam and the probe beam. The average power of pump beam is maintained at 40 mW while that of probe beam is 8 mW. The pump beam is used to generate THz pulse at the emitter which is composed of a photoconductive antenna (PCA) while the probe beam transmits and is controlled by a delay stage. In the system, the probe beam acts as a gated detector to monitor the temporal waveform of THz field. After being focalized and reflected by a lens and a mirror, the collimated THz pulse transmits the sample and then the sample-information-carried THz pulse reaches the silicon wafer, meeting the delayed probe beam. The optical signal is detected by a balance detector, amplified by a lock-in amplifier and finally processed by the LabVIEW software [18,19,20].

The interaction between water molecules leads to the formation of a complex multi-body system, which has strong absorption of terahertz waves. Therefore, attention must be paid while measuring water with THz-TDS. The first is that the thickness of water layer must be thin enough for the transmission measurement. In addition, the sample needs to be placed in a nitrogen atmosphere to eliminate the influence of water vapor. Besides, to obtain optimal signal it is important to place the sample at the focus.

The degree of attenuation of the THz radiation in the chip can be related to the flow pattern. The loop-pipe wall was pre-wetted by water and the flow monitoring experiment consisted of three types of measurements: the water volume flow-rate (*Q*), the pressure drops and the THz-TDS. The flow-rate was controlled accurately by the pre-set digital injection pump while the pressure drop was measured using two digital pressure gauges across the stream wise length of the channel, and the THz-TDS was recorded when the flow remained a stable state. In the experiment, to simplify the data processing, we ensured the THz wave transmitted the microchannel perpendicularly. The temperature and humidity were controlled at the range of 21.2–21.4 °C and 1.0–1.3%, respectively, in the experiments.

## 3. Results and Discussion

The flow monitoring experiment consisted of a recorded THz pulse waveform in the time domain and pressure drop at varying velocity intervals. By adjusting the parameters of the microsyringe pump, the flow-rate was controlled to range from 0 mL/min to 3.35 mL/min. Each of the waveforms represents a ‘snapshot’ of flowing performance at the flow-rate recorded. The empty PDMS chip was used as the reference measurement.

**Figure 2:**
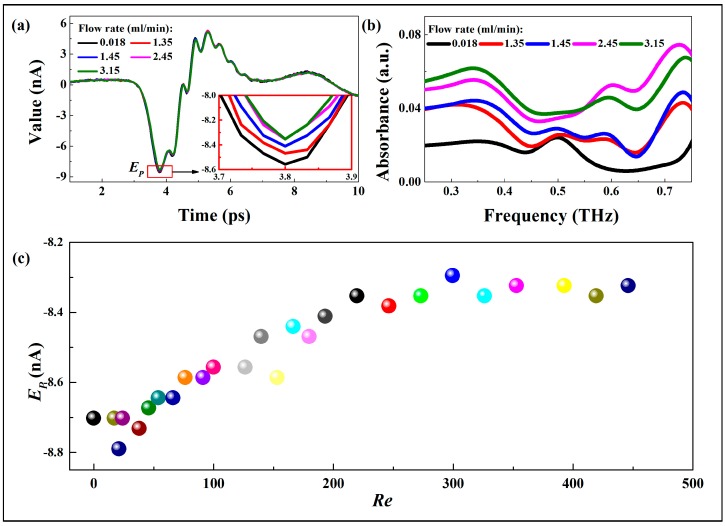
(**a**) Time domain trace of the THz pulse through the flow at different flow-rates and the partial enlarged detail (inset); (**b**) The absorbance curves against frequency for several flow rates; (**c**) Output THz peak trough intensities versus *Re* for single water flow.

Figure 2a shows the output THz-TDS of flows at several flow rates and the difference between peak trough intensities can be observed from the partial enlarged detail. The absorbances at these flow rates (Figure 2b) are calculated by a numerical Fast Fourier Transform (FFT). The FFT of the time-domain waveforms of reference and sample enables determination of the sample absorbance and permittivity et al., which are the basis of THz-TDS [21,22,23]. The absorbances (*A*) are calculated as a function of THz frequency (*ν*) using the following equation:

(2) $$A(\nu )=-\ln[E_{sample}(\nu )/E_{reference}(\nu )]$$

where *E_sample_* and *E_reference_* refers to the THz amplitude of sample and reference respectively [24].

Figure 2c shows the derived peak trough intensities at 27 Reynolds numbers. *Re* is a dimensionless parameter to determine if a flow is laminar or turbulent, and can be calculated as:

(3) $$Re=\frac{\rho vD_{H}}{\mu },v=\frac{Q}{S}$$

where *ρ* is the fluid density, *v* is the average flow velocity, and *μ* is the dynamic viscosity. The signals transmitting through the chip show the presence of peak trough intensity features at different *Re*. As expected, the signal loss increases with the increase of *Re*, but it seems to occur in two-phases which is consistent with the results in our previous research [14]: a first fast phase until *Re* reaches about 250, and next a second phase showing a slower signal loss. Then the question arises why, although the flow seems to be in the laminar range, there is such an inversion point in the *E_P_* changing along with *Re*. Therefore, the experimental results were further analyzed in the next section.

**Figure 3:**
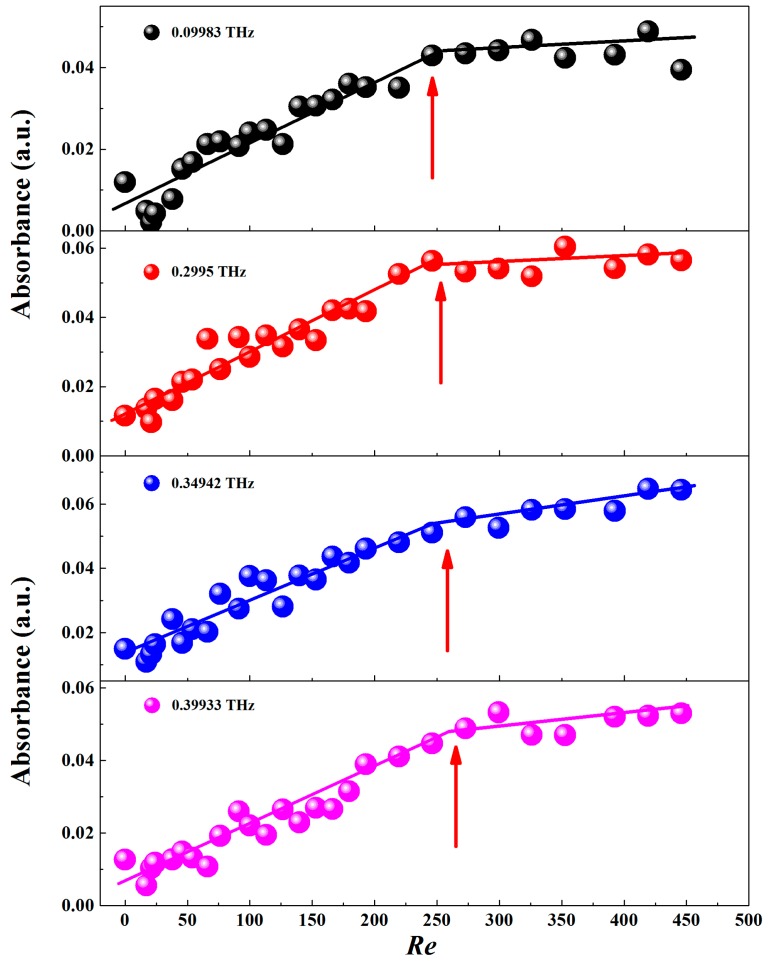
The THz absorbance as a function of *Re* at various frequencies.

In order to investigate the dependence of THz signal on *Re*, we extracted the absorbances at certain frequencies, as shown in Figure 3. It clearly shows that the higher the *Re* is, the stronger the absorbance is. Interestingly, regarding the dependence of *Re* on absorption in Figure 3, it is self-evident to approximate both linear relationships for the two stages with different slopes. In the first stage (the small *Re* region) there is an obvious increase of the absorbance. However, with an increase in *Re* (the second stage), the absorbance grows much more slowly. As per our understanding, it is believed that the slope change is caused by the early transition of flow pattern from laminar to transition flow in the rectangular microchannel which has been observed by some scholars [25].

**Figure 4:**
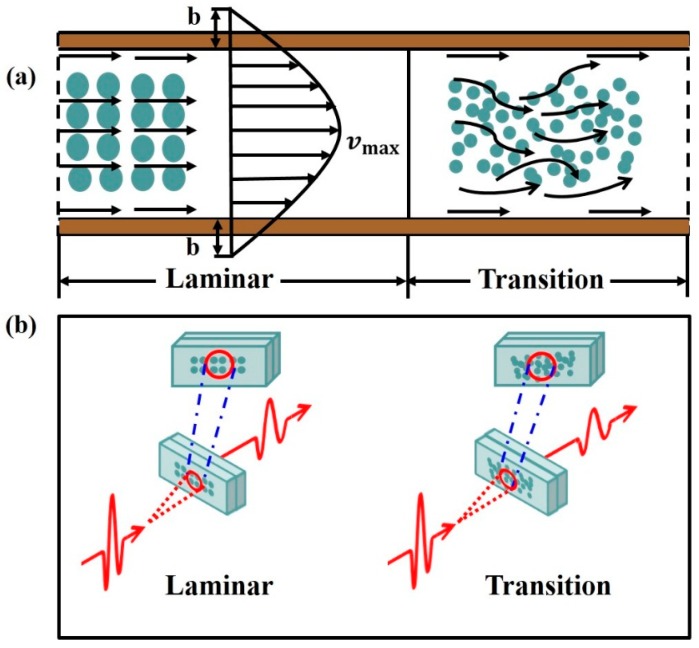
The schematic diagram for the interaction between THz wave and the flow. (**a**) The sketch map of laminar and transition; (**b**) Interaction between THz wave and the laminar and transition flow.

From the traditional theory, it is known that the internal flow undergoes a remarkable transition from laminar to transition and then a turbulent regime when the *Re* raises to certain values. The origin of turbulence and the accompanying transition from laminar to transition and to turbulent flow is of great importance to the studies in fluid mechanics. The possible flow patterns here are shown in Figure 4a. In the flow through the uniform straight microchannel at low *Re*, every fluid particle moves with a uniform velocity with velocity grades along vertical direction of fluids flow despite the existence of slippage caused by the hydrophobicity of channel surface. Due to the effect of viscous forces on the flow particles, the velocity of the particles near the wall is smaller than those in the center core. The flow is well ordered and the particles travel along neighboring layers in the straight microchannel (laminar flow). As the *Re* value increases, the number of particles increases and the maximum particle size reduces. However, for further increase of *Re*, the momentum exchange from different layers takes place, making strong mixing of the particles from the layers and the orderly pattern of flow cease to exist (transition flow) [26].

Figure 4b depicts the theoretical analysis for the experimental results. In laminar region, along with the increasing *Re*, the well-ordered small water particles in the detecting area will linearly increase the THz wave absorption. What’s more, with decreasing water particle size, the scattering effect may be enhanced as well, which would be another factor contributing to the THz absorption [13]. In transition region, the number of particles tend to be more while the maximum size tends to be smaller. However, the mixed particles from different layers reduce the augment of water to a certain degree in the test section, resulting in the slower growth in the THz wave absorbance. Our observation provides a sensitive way to monitor the flow characteristic of single phase water flow in the rectangular microchannel.

**Figure 5:**
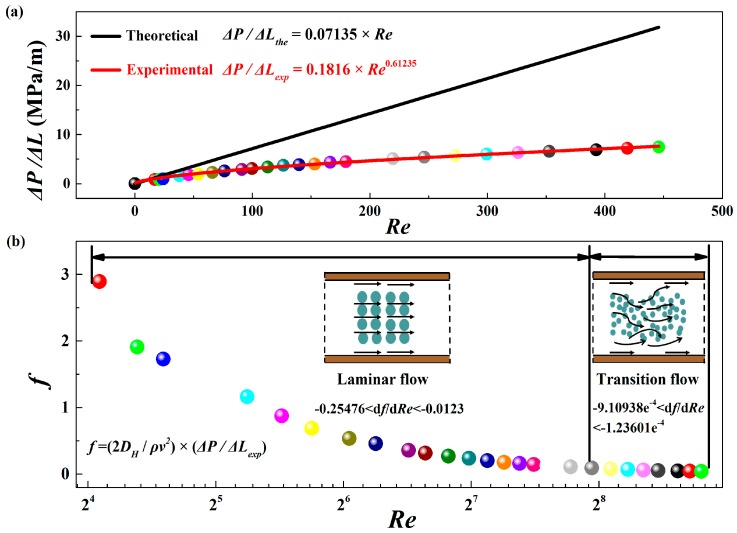
(**a**) The curve for the pressure gradient as a function of *Re*; (**b**) The flow resistance versus *Re* for the single flow in microchannel.

The most fundamental feature to discriminate the flow pattern is a noticeable change in the pattern of flow resistance (Darcy friction factor *f*). Therefore, the experimental pressure gradients (Δ*P*/Δ*L* in Figure 5a) were compared with the theoretical ones calculated by the Darcy-Weisbach Equation:

(4) $$\begin{array}{l} \Delta P/\Delta L=\frac{f\rho v^{2}}{2D_{H}} \end{array}$$

where Δ*L* is the channel length and the theoretical flow resistances can be obtained by:

(5) $$f=\frac{P_{O}}{Re}$$

for fully developed laminar flow in rectangular microchannel, the theoretical Poiseuille number *P_O_* can be computed numerically as the following equation given by Hartnett and Kostic [27]:

(6) $$P_{O}=96(1-1.3553\beta +1.9467\beta^{2}-1.7012\beta^{3}+0.9546\beta^{4}-0.2537\beta^{5})$$

in which *β* is the channel aspect ratio which must be less than 1, and the inverse should be taken if it is greater than 1.

It is found that the experimental pressure gradients indicate a significant departure from the theoretical predictions as the black line shows in Figure 5a. The departure is caused by the hydrophobicity of the microchannel surface. As channel size decreases, the hydrophobicity of microchannel surface strongly affects the lubrication and sliding friction of the flow [28,29] and plays a critical role in pressure drop across the channel [30,31,32]. The PDMS plate used in this experiment has not been surface modified further and thus the microfluidic chip remains hydrophobic in the experiment. Because of the wall hydrophobicity, apparent water slip was observed, resulting from the obvious decrease of pressure gradients. Different from the linear relationship between the pressure gradient required to force liquid through the microchannel and *Re* in conventional laminar flow theory, a nonlinear relationship (Δ*P*/Δ*L_exp_* = 0.1816 × *Re*^0.16235^) is observed here, which is caused by the turbulent flow.

Then some valuable information can be gathered by analyzing the variation tendency of experimental *f*. *f*, as depicted in Figure 5b, is calculated by the above-mentioned Darcy-Weisbach equation. For smaller *Re*, *f* decreases rapidly along with the rising *Re*, which is the characteristic of laminar flow resistance. By contrast, for larger *Re*, *f* almost keeps unchanged, which is the characteristic of transition flow resistance [33,34]. To make sure the value of critical *Re*, the differential values of *f* (d*f*/d*Re*) were derived. −0.25476 < d*f*/d*Re* < −0.0123 when *Re* is less than 250 while d*f*/d*Re* approaches to zero when *Re* is greater than 250, which means *f* decreases sharply until *Re* reaches to about 250. Overall, from the above analysis, it can be concluded that for the microchannel, there is an early transition from laminar to transition flow at *Re* about 250.

The need for reliable and effective design and fabrication of such microdevices has been the driving force behind an extensive effort to understand the fluid flow fundamentals on the microscale [35]. It has been reported that the smaller the typical length scale of the microfluid flow is, the smaller the critical *Re* from laminar to fully developed turbulent flow becomes compared with the ordinary channel flow [36,37]. We have employed THz-TDS to investigate the flow characteristics of single phase water flow in a rectangular PDMS microchannel under the condition of varying water flow-rates in the experiment. It is found that the THz peak trough intensities and the absorbances of the flowing water can accurately determine the transition from laminar to transition flow. In the laminar region, owing to the good order of the abundant small water particles, the THz parameters increase linearly with the increasing *Re* while in the transition region, the THz parameters increase much more slowly because of the mixed particles from different layers in the test section. By analyzing the pressure gradient and the flow resistance, the results are further demonstrated in theory. This approach can serve as a novel and practicable technique to determine the flow characteristics of single water flow. Further studies should be conducted to comprehend more about the potential flow mechanisms in microfluidic flow based on THz-TDS.

## 4. Conclusions

The flow characteristics of water with different flow-rates in a rectangular microchannel whose hydraulic diameter was 80 μm were investigated experimentally and theoretically. It is observed that for smaller *Re* (less than 250), the THz peak trough intensities as well as the THz wave absorbances of the flow increases were sharply compared with larger *Re*, which indicates that the transition from laminar to transition flow takes place at an early *Re* compared with the conventional theory, and that the THz-TDS is indeed an effective tool for the study of flow characteristics. Based on the measured pressure drop, the flow resistances were derived as well. It is observed that for smaller *Re*, *f* decreases much rapidly than that for larger *Re*, which further proves the early transition of the flow pattern. As an initial effort, this study used pure water as an idealized case to show the potential of THz-TDS in studying the flow in microchannels, and the mixture of water, oil and gas should be considered in further studies to produce results closer to practical situations.

## Abbreviations

The following abbreviations are used in this manuscript:

THz-TDS	Terahertz time domain spectroscopy
Re	Reynolds number
microPIV	Microscopic particle image velocimetry
PDMS	Polydimethylsiloxane
DOCT	Doppler optical coherence tomography
PTFE	Polytetrafluoroethylene
FFT	Fast Fourier Transform
